# The Efficacy of Pegylated Liposomal Doxorubicin-Based Neoadjuvant Chemotherapy in Breast Cancer: A Retrospective Case-Control Study in Taiwan

**DOI:** 10.1155/2020/5729389

**Published:** 2020-04-28

**Authors:** Chih-Chiang Hung, Youngsen Yang, I-Chen Tsai, Chiann-yi Hsu, Chia-Hua Liu, Jie-Ru Yang

**Affiliations:** ^1^No. 1650, Taiwan Boulevard Section 4, Xitun District, Division of Breast Surgery, Department of Surgery, Taichung Veterans General Hospital, 40705 Taichung, Taiwan; ^2^No. 1018, Section 6 Taiwan Boulevard, Shalu District, Department of Applied Cosmetology, College of Human Science and Social Innovation, Hungkuang University, 43302 Taichung, Taiwan; ^3^No. 1650, Taiwan Boulevard Section 4, Xitun District, Division of Hematology-Oncology, Department of Internal Medicine, Taichung Veterans General Hospital, 40705 Taichung, Taiwan; ^4^No. 91, Hsueh-Shih Road, North District, Internal Medicine, College of Medicine, China Medical University, 40402 Taichung, Taiwan; ^5^No. 91, Hsueh-Shih Road, North District, Graduate Institute of Biomedical Sciences, China Medical University, 40402 Taichung, Taiwan; ^6^No. 1650, Taiwan Boulevard Section 4, Xitun District, Biostatistics Task Force, Taichung Veterans General Hospital, 40705 Taichung, Taiwan

## Abstract

Breast cancer is a global issue regarding women's health, and high incident rates remain in the Taiwanese female population. Chemotherapy, using anthracycline-based chemotherapeutic agents in neoadjuvant settings, has been introduced as a promising new therapeutic option for treatment of invasive breast cancer. Set apart from conventional anthracycline regimens such as epirubicin, pegylated liposomal doxorubicin (Lipo-Dox®, PLD) was introduced for providing a justifiable treatment effect, while offering a favorable toxicity profile for breast cancer patients in a metastatic setting. However, the efficacy of PLD in neoadjuvant settings for breast cancer patients has not yet been sufficiently reported. This study aims to investigate the efficacy of PLD-based neoadjuvant chemotherapy in breast cancer patients using a retrospective matched case-control study. A total of 183 PLD cases and 183 epirubicin-based controls were included after a 1 : 1 ratio case-control matching procedure was held, according to the matching criteria. These criteria included the patient's preoperative clinical stage, molecular subtype, chemotherapy regimen with taxanes prior to surgery, and histological grade. All data were collected according to an institutional review board approved protocol. The study results reported that the PLD and epirubicin groups both obtained similar outcomes in pathologic complete response (pCR), recurrence, and overall survival rate with no statistically significant differences. Overall, the study results demonstrate that PLD-based neoadjuvant chemotherapy offers a similar effect of treatment with a favorable toxicity profile within the study follow-up duration, when compared with conventional epirubicin-based neoadjuvant chemotherapy.

## 1. Introduction

Chemotherapy in neoadjuvant settings has been introduced as a new therapeutic approach in the treatment of invasive breast cancer for the purpose of eliminating the invasive tumor cells and occult tumor lesions, through the use of anthracycline-based chemotherapeutic agents prior to surgery [1–3]. Liposomal-encapsulated doxorubicin is commonly used in breast cancer therapeutics [4, 5]. The liposomal-encapsulated characteristics of pegylated liposomal doxorubicin (PLD) are advantageous in that it offers a reduction in chemotherapy-related toxicity, particularly because the lower cardiotoxicity profile of PLD allows for a wider usage in breast cancer therapeutics [6–9]. Previous studies have revealed that PLD is one of the promising chemotherapy regimens for breast cancer patients, apart from conventional anthracycline-based chemotherapeutic agents [4, 5, 10–12].

The response to neoadjuvant treatment is usually used to determine the efficacy of neoadjuvant therapeutics and the chemosensitivity of tumor lesions. Breast cancer patients who have achieved pathologic complete response (pCR), including having no remaining invasive tumor in the breast and lymph nodes, were more likely to obtain better outcomes when compared to non-pCR subjects [13, 14]. Although pCR could be considered as a primary endpoint for neoadjuvant therapeutic efficacy determination, pCR is still not a definite surrogate endpoint for long-term breast cancer survival. The prognosis outcome, including disease progression and overall survival, is considered as the surrogate endpoints regarding long-term survival for cancer patients [15, 16]. Nevertheless, previous studies have revealed that pCR is positively associated with long-term prognostic outcome enhancement in high-risk or aggressive subtypes of breast cancer [17, 18]. Hence, pCR is considered to be the primary endpoint for breast cancer patients who have received neoadjuvant therapy and then allowing for further estimates regarding long-term survival using disease progression and overall survival.

Breast cancer remains a global health concern and a major women's health issue, with a consistently high incidence rate in the Taiwanese female population being reported since 2007 [19, 20]. Along with the increased incidence rate, breast cancer resulting in both morbidity and mortality has become a major health burden to female Taiwanese residents. Hence, more effective therapeutic options are desired in order to prolong relapse intervals and survival times for breast cancer patients. Although PLD chemotherapeutic agents have been reported to obtain warranted treatment effects for breast cancer in metastatic settings, the efficacy in neoadjuvant settings for breast cancer patients has not yet been sufficiently reported. The tumor characteristics, molecular subtypes, and additional target therapeutics have been reportedly associated with survival outcomes in neoadjuvant settings [21–23]. Therefore, this study aims to investigate the efficacy of PLD-based neoadjuvant chemotherapy in breast cancer patients using a retrospective matched case-control study. The breast cancer patients who had previously received a conventional anthracycline-based chemotherapeutic agent named epirubicin were used as controls in order to compare the efficacy and toxicity profile with a PLD group, after matching using the study defined matching criteria.

## 2. Materials and Methods

### 2.1. Datasets

The PLD group included the patients who had received PLD 35 mg/m^2^ plus cyclophosphamide (600 mg/m^2^, every 21 days for 4 cycles) and/or taxane regimen including docetaxel (75 mg/m^2^, every 21 days for 4 cycles) or paclitaxel (80 mg/m^2^, weekly for 12 cycles) in neoadjuvant settings. However, the epirubicin group included the patients who had received epirubicin 90 mg/m^2^ plus cyclophosphamide 600 mg/m^2^ every 21 days for 4 cycles and/or taxane regimen including docetaxel (75 mg/m^2^, every 21 days for 4 cycles) or paclitaxel (80 mg/m^2^, weekly for 12 cycles) in neoadjuvant settings. A total of 239 patients in the PLD group, along with 344 patients in the epirubicin group, were enrolled in a 1 : 1 ratio case-control matching procedure based in part on data taken from the Taichung Veterans General Hospital Research Database and managed by the Clinical Informatics Research & Development Center of Taichung Veterans General Hospital (Registered number CE19346A) (the interpretation and conclusions contained herein do not represent to those of Taichung Veterans General Hospital).

The matching criteria included the patient's preoperative clinical stage (stages IA, IIA, IIB, IIIA, IIIB, and IIIC), histology grade (grades 1-2, 3, and unknown), molecular subtype (triple negative breast cancer (TNBC), human epidermal growth factor receptor 2 positive breast cancer (Her2-positive subtype), and luminal subtype (Her2 negative)), and neoadjuvant chemotherapy regimen (with or without taxane). All clinical data of enrolled patients was retrospectively collected from the cancer registry database and included diagnosed age, preoperative clinical stage, histology grade, tumor size, positive number of axillary lymph nodes, histological type, molecular status, treatment options, and follow-up status. The adverse effects (AEs) caused by either PLD or the epirubicin regimen were monitored using the laboratory examination records documented in each patient's medical record, including leukocytes, absolute neutrophil count, hemoglobin, and platelet measurements for blood function monitoring, along with the enzymes aspartate transaminase (AST) and alanine aminotransferase (ALT) measurements for hepatic function monitoring. The patients with no invasive tumor in the breast and found to be node-negative after definite surgery were considered to have obtained total pCR [24–26]. The study endpoints included disease progression and all-cause mortality. Breast cancer patients with disease progression, including recurrence or metastasis, were considered recurrence subjects, and all-cause mortality indicates the subjects who died within the study follow-up period. All patients were tracked from January 2009 to December 2017.

A total of 366 patients were included after 1 : 1 ratio case-control matching procedure was performed according to the matching criteria, which included the patient's preoperative clinical stage, molecular subtype, neoadjuvant chemotherapy regimen with taxane prior to surgery, and histological grade. As shown in Table 1, we identified 12 (6.6%) patients with stage I cancer, 141 (77.0%) with stage II, and 30 (16.4%) with stage III in diagnosed preoperative clinical staging among the PLD (183 patients) and epirubicin (183 patients) groups. There were 77.6% patients who had received a chemotherapy regimen with taxane prior to surgery. According to the molecular subtype characteristics, 170 (46.4%) patients were luminal subtypes (Her2 negative), 130 (35.5%) were Her2-positive subtypes (about 18 patients in each group had received neoadjuvant target therapy with pertuzumab and trastuzumab, while around 50 patients in each group had been treated with trastuzumab only), and 66 (18.0%) were TNBC subtypes. According to the histological grade, 58.5% patients were low grade (grades 1 and 2), 37.7% patients were high grade (grade 3), and 3.8% patients had unknown grade characteristics.

### 2.2. Statistical Analysis

The baseline characteristics, neoadjuvant chemotherapy response, adjuvant treatment options, study endpoints, and laboratory measurements of the included breast cancer patients were summarized in both number and percentages appropriately. The differences between the PLD and epirubicin groups were evaluated using the chi-square test or Fisher's exact test. Continuous variables were presented as a mean and standard deviation (SD), with normality distribution determined by the Kolmogorov–Smirnov test and compared using the Mann–Whitney *U* test. A Cox proportional hazard regression analysis was performed to determine the impact of the various neoadjuvant regimen effects and covariates on overall survival (OS) with the independent variables with a univariate of *p* < 0.1 included in the multivariate model. The association was expressed as a hazard ratio (HR), with a 95% confidence interval (CI). The overall survival rate and cumulative recurrence rate of breast cancer patients in the study follow-up interval were demonstrated using the Kaplan–Meier method, and the differences in the survival status and recurrence between the PLD and epirubicin groups were estimated using the log-rank test. All *p* values were two-sided, and a *p* value less than 0.05 was considered statistically significant. All statistical analyses were performed using the Statistical Package for the Social Science (IBM SPSS version 22.0; International Business Machines Corp, New York, USA).

## 3. Results and Discussion

Table 1 presents the distribution of baseline characteristics of the included patients by both the PLD and epirubicin regimens used in neoadjuvant settings. The mean age of the PLD group was 49.7 ± 8.6 years, while the mean age of the epirubicin group was 49.3 ± 9.7 years. No statistical significance was found in the baseline characteristics between both groups including tumor size, histological type, and molecular status. Sixty-eight (37.2%) PLD subjects and 85 (46.4%) epirubicin subjects received partial mastectomy after the completion of neoadjuvant chemotherapy. In the PLD group, 138 (75.4%) subjects received radiotherapy, 126 (68.9%) subjects received hormone therapy, and 70 (38.3%) subjects received target therapy. In the epirubicin group, 133 (72.7%) subjects received radiotherapy, 132 (72.1%) subjects received hormone therapy, and 70 (38.3%) subjects received target therapy. Both groups showed similar distribution in their therapy options. Thirty-three (18.0%) PLD subjects and 36 (19.7%) epirubicin subjects obtained pCR after neoadjuvant therapy, without any statistical significance between the two groups (*p*=0.789). The pCR in each molecular subtype was 10 (5.9%) patients in luminal subtype, 45 (34.6%) patients in Her2-positive subtype, and 14 (21.2%) patients in TNBC. During the study follow-up duration, 16 (8.7%) PLD subjects and 20 (10.9%) epirubicin subjects experienced recurrence events. In addition, 25 (13.7%) PLD subjects and 14 (7.7%) epirubicin subjects died within the study follow-up interval.

During a median follow-up of 3.0 years, 16 recurrence subjects (8.7%) in the PLD group and 20 recurrence subjects (10.9%) in the epirubicin group were observed.  Table 2 demonstrates the distribution of baseline characteristics of neoadjuvant breast cancer patients according to the overall survival status. The PLD and epirubicin groups showed no significant differences in proportion between the alive and died groups. However, the pCR subjects showed a significantly lower proportion in the died group (died vs. alive: 2.6% vs. 20.8%, *p*=0.011). In addition, patients reaching advanced preoperative clinical stages (IIIA-IIIB, died vs. alive: 2.6–15.4% vs. 0.3–12.2%, *p*=0.016), TNBC subjects (died vs. alive: 30.8% vs. 16.5%, *p*=0.024), high grade (died vs. alive: 66.7% vs. 34.3%, *p* < 0.001), and total mastectomy (died vs. alive: 74.4% vs. 56.3%, *p*=0.024) were at a significantly higher proportion in the died group. In addition, the died subjects experienced a significantly greater tumor size (died vs. alive: 50.5 ± 30.3 vs. 33.4 ± 18.4, *p* < 0.001), compared to the living group. The majority of died patients was due to progression of breast cancer. Three patients died in the PLD group due to other causes, involving infection, intracerebral hemorrhage, and leukemia. Moreover, a lower percentage of estrogen receptor (ER) or progesterone receptor (PR) status, i.e., less than 10–20%, was labeled as being a luminal subtype but that was considered to have a lower efficacy for adjuvant hormone therapy. In the PLD group, the died patients had a relatively lower ER or PR status compared with the epirubicin group.

**Figure 1:**
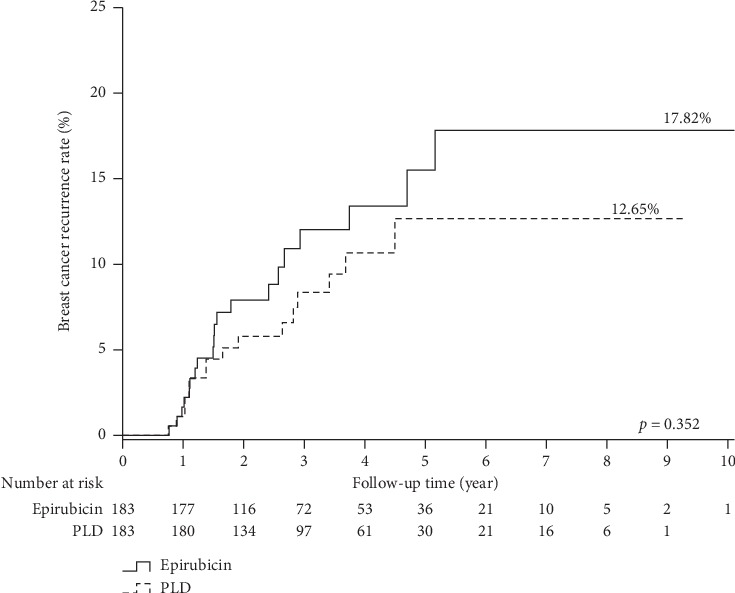
Cumulative recurrence estimates of breast patients treated with PLD versus epirubicin neoadjuvant chemotherapy.

Figure 1 visualizes the cumulative recurrence estimates of breast cancer patients treated with PLD-based versus epirubicin-based neoadjuvant chemotherapy. The cumulative breast cancer recurrence rate computed by Kaplan–Meier methods was 12.65% and 17.82% (log-rank test; *p* value = 0.352) in the PLD and epirubicin groups, respectively.

**Figure 2:**
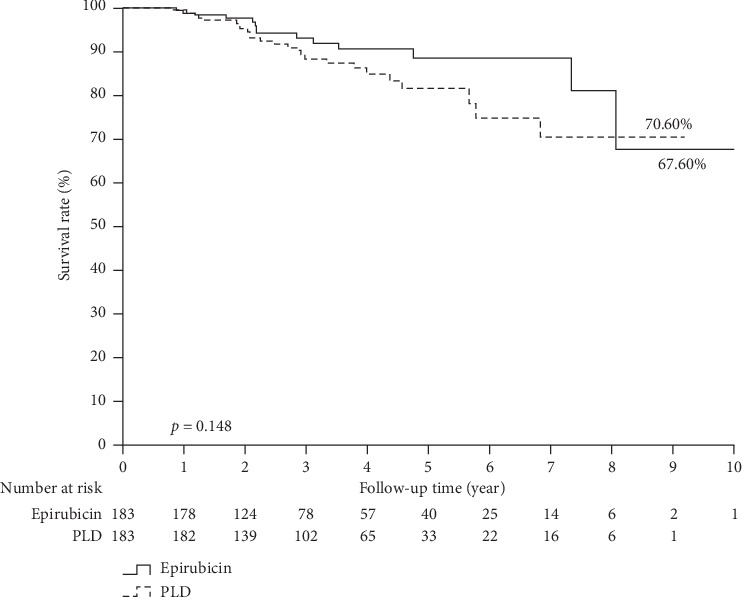
Overall survival estimates of breast cancer patients treated with PLD versus epirubicin neoadjuvant chemotherapy.

Figure 2 illustrates the overall survival estimates of breast cancer patients treated with PLD-based versus epirubicin-based neoadjuvant chemotherapy. In overall survival estimation, 25 (13.7%) PLD subjects and 14 (7.7%) epirubicin subjects had died within the follow-up interval. According to the Kaplan–Meier estimation, the overall survival rate was 70.6% and 67.6% (log-rank test; *p* value = 0.148) in the PLD and epirubicin groups, respectively. Overall, the study results show no statistical difference between the PLD and epirubicin groups in both disease progression and overall survival rate.

Table 3 depicts the overall survival analysis results of neoadjuvant treatment effects and their associated covariates using the Cox proportional hazard regression method. In univariate analysis, breast cancer patients with TNBC (HR = 3.14, 95% CI = 1.38–7.13, *p*=0.006), high grade (HR = 3.95, 95% CI = 1.99–7.83, *p* < 0.001), and greater tumor size (HR = 1.02, 95% CI = 1.01–1.03, *p* < 0.001) could have their overall mortality risk increased. The multivariate analysis shows consistently significant results in high grade (HR = 2.71, 95% CI = 1.28–5.76, *p*=0.009) and greater tumor size (HR = 1.02, 95% CI = 1.01–1.03, *p* < 0.001). Furthermore, the multivariate analysis results indicate that patients who received a regimen with taxane prior to surgery (HR = 2.4, 95% CI = 1.09–5.25, *p*=0.029) could obtain a higher overall mortality risk. Moreover, patients with pCR (HR = 0.12, 95% CI = 0.02–0.88, *p*=0.037) could obtain overall survival benefits in multivariate analysis. Similar with the findings mentioned above, the treatment effects between PLD and epirubicin showed no statistically significant difference in overall survival in both univariate and multivariate analyses.

Table 4 summarizes the laboratory measurements for adverse effects monitoring, as reported in the PLD and epirubicin groups. For blood function monitoring, hemoglobin count in the PLD group showed a significantly higher proportion of ≥12 grams per deciliter compared to the epirubicin group (PLD vs. epirubicin: 28.2% vs. 7.5%, *p* < 0.001). Otherwise, the leukocytes, absolute neutrophil count, and platelet measurements showed no significant difference between the two groups. The hepatic function monitoring measurements, including AST and ALT, also showed no significant difference between the PLD and epirubicin groups, with most of the patients obtaining less than or equal to 87.5 units per liter in AST and ALT levels.

This is a retrospective matched case-control study to observe neoadjuvant PLD treatment effects compared with conventional epirubicin therapeutics for breast cancer patients in Taiwan. The study results reveal a warranted treatment effect in the PLD group compared with the conventional epirubicin group in neoadjuvant settings. Moreover, the PLD group obtained a lower toxicity profile, particularly in hemoglobin count. Consistent with the previous findings from PLD studies in breast cancer, the PLD and epirubicin groups showed similar effects in pathological response after neoadjuvant therapy, disease-free survival, and overall survival [27–30]. Moreover, several prospective studies also have demonstrated that they could provide potentially similar efficacy and manageable toxicity in the neoadjuvant treatment of breast cancer [12, 31, 32].

The current study indicates that mortality risk is associated with a high histological grade, larger tumor size, and TNBC in overall survival follow-up. The larger tumor size and high grade in histological proliferation of breast cancer are known risk factors for poor survival outcomes [23, 33, 34]. The TNBC subtype has been previously reported to be associated with a poor prognosis outcome, while the findings of current study also indicate that TNBC subjects were associated with an increased mortality risk [35]. The study results also indicate that the patients who received a chemoregimen with taxane prior to surgery could obtain a higher mortality risk, which may be due to the taxane regimen being particularly important for TNBC, Her2-positive subtype, and those who have lymph node-positive invasive breast cancer [36, 37]. In general, lymph node-positive invasive breast cancer was more likely to obtain a poor survival outcome compared to lymph node-negative breast cancer [38].

Moreover, pCR subjects were more likely to obtain a prolonged overall survival in the current findings, which is consistent with previous research findings [39, 40]. The PLD and epirubicin groups still obtained similar treatment effects in both univariate and multivariate regression analysis after a matched case-control design, which demonstrates the warranted efficacy of PLD for breast cancer in neoadjuvant settings. In addition, the laboratory measurements for adverse effects indicated that the PLD group could obtain favorable monitoring outcomes compared with the epirubicin group.

In both groups, most causes of mortality were advanced stage disease, TNBC, and stable to progressive disease after neoadjuvant treatment in the Her2-positive subtype. To improve the outcome of patients with residual invasive Her2-positive breast cancer, further treatment involving anti-Her2 therapy, such as trastuzumab emtansine, may be an option [36, 37]. If experiencing residual invasive disease after neoadjuvant chemotherapy in the TNBC subtype, adjuvant capecitabine could be considered for improving a patient's outcome [36, 37]. The National Health Insurance of Taiwan have reimbursement of taxane for the breast cancer patients with lymph node metastasis and TNBC subtype. Hence, most of patients with clinical stages I and IIA tended to not receive taxane due to the extra self-payment. In general, patients with lymph node metastasis and TNBC subtype were more likely to achieve poor survival outcome, wherefore the patients in our study who received taxane having a worse prognosis outcome were predictable.

Several limitations should be mentioned regarding the current study. First, the retrospective nature of the current study limited the inclusion of potential covariates for breast cancer prognosis. Second, all patients included were from a single center institution. Third, toxicity profile monitoring was dependent upon the retrospective medical database, with cardiac toxicities and various subjective adverse events such as nausea and vomiting, being difficult to subsequently include in the current study. These mentioned limitations could restrict the generalization and validity of our study findings. However, the current study still offers a valuable contribution towards the evaluation of treatment effects of PLD and conventional epirubicin in neoadjuvant settings, using a retrospective stringent matched case-control study. The study results demonstrate that the warranted treatment effects of PLD compared to epirubicin in neoadjuvant settings, along with PLD therapeutics, may offer more favorable laboratory monitoring outcomes compared to the conventional epirubicin regimen.

## 4. Conclusion

This study reported on the efficacy of PLD-based neoadjuvant chemotherapy in breast cancer patients, compared to conventional epirubicin-based neoadjuvant chemotherapy, using stringent case-control matching criteria. Compared with the conventional epirubicin-based neoadjuvant chemotherapy, the study results indicate that PLD-based neoadjuvant chemotherapy offers similar treatment effects with a favorable toxicity profile within the follow-up duration. Thence, PLD could be a viable option for breast cancer patients in neoadjuvant settings, particularly for elderly patients and those with cardiovascular comorbidity. Further evaluation regarding PLD therapeutics involving a larger sample size and longer follow-up duration using a prospective study is still desired in order to further investigate the advantages of PLD in neoadjuvant settings for Taiwan's female breast cancer population.

## Figures and Tables

**Table 1:** Baseline characteristics of breast cancer patients according to different neoadjuvant chemotherapeutic regimens.

Characteristics	Epirubicin (*n* = 183)		PLD (*n* = 183)		Total (*n* = 366)		*p* value
	*N*	(%)	*n*	(%)	*n*	(%)	
Clinical stage f							1.000
IA	12	(6.6)	12	(6.6)	24	(6.6)	
IIA	63	(34.4)	63	(34.4)	126	(34.4)	
IIB	78	(42.6)	78	(42.6)	156	(42.6)	
IIIA	23	(12.6)	23	(12.6)	46	(12.6)	
IIIB	6	(3.3)	6	(3.3)	12	(3.3)	
IIIC	1	(0.5)	1	(0.5)	2	(0.5)	
Molecular subtype							1.000
Luminal type (Her2 negative)	85	(46.4)	85	(46.4)	170	(46.4)	
Her2 positive	65	(35.5)	65	(35.5)	130	(35.5)	
TNBC	33	(18.0)	33	(18.0)	66	(18.0)	
Chemoregimen with taxane before surgery	142	(77.6)	142	(77.6)	284	(77.6)	1.000
Histological grade							1.000
Grade 1-2 (well/moderately differentiated)	107	(58.5)	107	(58.5)	214	(58.5)	
Grade 3 (poorly differentiated)	69	(37.7)	69	(37.7)	138	(37.7)	
Grade 9 (unknown)	7	(3.8)	7	(3.8)	14	(3.8)	
Age (mean ± SD)^#^	49.3	±9.7	49.7	±8.6	49.5	±9.2	0.789
Tumor size (mm) (mean ± SD)^#^	35.6	±19.5	34.8	±21.8	35.2	±20.6	0.239
Surgery							0.090
Partial mastectomy	85	(46.4)	68	(37.2)	153	(41.8)	
Total mastectomy	98	(53.6)	115	(62.8)	213	(58.2	
Therapy							
Radiotherapy (RT)	133	(72.7)	138	(75.4)	271	(74.0)	0.633
Hormone therapy (HT)	132	(72.1)	126	(68.9)	258	(70.5)	0.567
Target therapy (TT)	70	(38.3)	70	(38.3)	140	(38.3)	1.000
Recurrence	20	(10.9)	16	(8.7)	36	(9.8)	0.598
Died	14	(7.7)	25	(13.7)	39	(10.7)	0.090
Pathologic complete response (pCR)	36	(19.7)	33	(18.0)	69	(18.9)	0.789

Chi-square test. ^f^Fisher's exact test. ^#^Mann–Whitney *U* test.

**Table 2:** Overall survival by baseline characteristics.

Characteristics	Alive (*n* = 327)		Died (*n* = 39)		*p* value
	*n*	(%)	*n*	(%)	
Age (mean ± SD)^#^	49.3	±9.3	51.5	±7.8	0.123
Tumor size (mm) (mean ± SD)^#^	33.4	±18.4	50.5	±30.3	<0.001^*∗∗*^
Neoadjuvant chemotherapeutic regimen					0.090
Epirubicin-based	169	(51.7)	14	(35.9)	
PLD-based	158	(48.3)	25	(64.1)	
Molecular subtype					0.024^*∗*^
Luminal type (Her2 negative)	159	(48.6)	11	(28.2)	
Her2 positive	114	(34.9)	16	(41.0)	
TNBC	54	(16.5)	12	(30.8)	
Histological grade f					<0.001^*∗∗*^
Grade 1-2 (well/moderately differentiated)	202	(61.8)	12	(30.8)	
Grade 3 (poorly differentiated)	112	(34.3)	26	(66.7)	
Grade 9 (unknown)	13	(4.0)	1	(2.6)	
Pathologic complete response (pCR) f	68	(20.8)	1	(2.6)	0.011^*∗*^
Chemoregimen with taxane before surgery	257	(78.6)	27	(69.2)	0.262
Clinical stage f					0.016^*∗*^
IA	24	(7.3)	0	(0.0)	
IIA	116	(35.5)	10	(25.6)	
IIB	138	(42.2)	18	(46.2)	
IIIA	40	(12.2)	6	(15.4)	
IIIB	8	(2.4)	4	(10.3)	
IIIC	1	(0.3)	1	(2.6)	
Surgery					0.046^*∗*^
Partial mastectomy	143	(43.7)	10	(25.6)	
Total mastectomy	184	(56.3)	29	(74.4)	
With radiotherapy	240	(73.4)	31	(79.5)	0.531

Chi-square test. ^f^Fisher's exact test. ^#^Mann–Whitney *U* test. ^*∗*^*p* < 0.05; ^*∗∗*^*p* < 0.01.

**Table 3:** Cox proportional hazard regression model of overall survival.

Characteristics	Univariate analysis		Multivariate analysis	
	HR (95% CI)	*p*	HR (95% CI)	*p*
PLD group vs. epirubicin group	1.62 (0.84–3.11)	0.152	1.36 (0.69–2.68)	0.373
Pathologic complete response (pCR) vs. non-pCR	0.14 (0.02–1.04)	0.055	0.12 (0.02–0.88)	0.037
Matching criteria				
Preoperative clinical stage (III vs. I-II)	1.75 (0.86–3.54)	0.121	0.78 (0.32–1.93)	0.596
Molecular subtype				
Luminal type (Her2 negative)	1.00		1.00	
Her2 positive	1.96 (0.91–4.23)	0.087	1.81 (0.8–4.13)	0.157
TNBC	3.14 (1.38–7.13)	0.006	2.25 (0.87–5.82)	0.096
Chemoregimen before surgery with taxane vs. without taxane	1.96 (0.91–4.23)	0.086	2.40 (1.09–5.25)	0.029
Histological grade (III vs. I-II)	3.95 (1.99–7.83)	<0.001	2.71 (1.28–5.76)	0.009
Age	1.04 (1.00–1.07)	0.063	1.01 (0.98–1.05)	0.532
Tumor size (mm)	1.02 (1.01–1.03)	<0.001	1.02 (1.01–1.03)	<0.001

**Table 4:** Laboratory measurements for adverse effects monitoring reported in PLD and epirubicin groups.

Measurements	Total (*n* = 366)		PLD (*n* = 183)		Epirubicin (*n* = 183)		*p*
	*N*	(%)	*N*	(%)	*n*	(%)	
Leukocytes (*μ*L)							0.606
≥3500	208	(59.8)	105	(60.3)	103	(59.2)	
≥3000–3500	56	(16.1)	31	(17.8)	25	(14.4)	
≥2000–3000	69	(19.8)	33	(19.0)	36	(20.7)	
≥1000–2000	13	(3.7)	4	(2.3)	9	(5.2)	
<1000	2	(0.6)	1	(0.6)	1	(0.6)	

Absolute neutrophil count (mm^3^)							0.510
≥2000	192	(55.2)	95	(54.6)	97	(55.7)	
≥1500–2000	82	(23.6)	44	(25.3)	38	(21.8)	
≥1000–1500	48	(13.8)	26	(14.9)	22	(12.6)	
≥500–1000	18	(5.2)	6	(3.4)	12	(6.9)	
<500	8	(2.3)	3	(1.7)	5	(2.9)	

Hemoglobin (g/dl)							<0.001
≥12	62	(17.8)	49	(28.2)	13	(7.5)	
≥10–12	195	(56.0)	91	(52.3)	104	(59.8)	
≥8–10	76	(21.8)	28	(16.1)	48	(27.6)	
≥6.5–8	12	(3.4)	5	(2.9)	7	(4.0)	
<6.5	3	(0.9)	1	(0.6)	2	(1.1)	

Platelets (*μ*L)							0.644
≥150000	339	(97.4)	169	(97.1)	170	(97.7)	
≥50000-75000	6	(1.7)	4	(2.3)	2	(1.1)	
≥1000-50000	2	(0.6)	1	(0.6)	1	(0.6)	
<10000	1	(0.3)	0	(0.0)	1	(0.6)	

AST (U/L)							0.745
≤87.5	324	(93.1)	161	(92.5)	163	(93.7)	
>87.5–100	7	(2.0)	3	(1.7)	4	(2.3)	
>100–200	13	(3.7)	7	(4.0)	6	(3.4)	
>200–800	4	(1.1)	3	(1.7)	1	(0.6)	

ALT (U/L)							0.490
≤87.5	294	(84.5)	145	(83.3)	149	(85.6)	
>87.5–100	11	(3.2)	8	(4.6)	3	(1.7)	
>100–200	32	(9.2)	16	(9.2)	16	(9.2)	
>200–800	11	(3.2)	5	(2.9)	6	(3.4)	

*p* values are estimated using Fisher's exact test.

## Data Availability

The cancer registry data used to support the findings of this study have not been made available because of the regulation of institute.
